# Automated frailty scores: towards clinical implementation

**DOI:** 10.18632/aging.204815

**Published:** 2023-06-08

**Authors:** Jonathan K. L. Mak, Dorota Religa, Juulia Jylhävä

**Affiliations:** 1 Department of Medical Epidemiology and Biostatistics, Karolinska Institutet, Stockholm, Sweden; 2 Division of Clinical Geriatrics, Department of Neurobiology, Care Sciences and Society, Karolinska Institutet, Stockholm, Sweden; 3 Theme Inflammation and Aging, Karolinska University Hospital, Huddinge, Sweden; 4 Faculty of Social Sciences (Health Sciences) and Gerontology Research Center (GEREC), University of Tampere, Tampere, Finland

**Keywords:** frailty, electronic health records, electronic frailty index, deficit accumulation, screening, geriatrics

Frailty is a state of physiological decline across multiple systems. It is common among older adults and is a strong predictor of mortality and other adverse outcomes independent of age. Given its public health burden especially during the COVID-19 pandemic, increasing efforts have been made in recent years to develop frailty assessment tools that could allow early identification and management of frail individuals. Although there is still no consensus on how we can best measure frailty, the Clinical Frailty Scale (CFS) is one of the most frequently adopted frailty measures in clinical settings. It is a simple, rapid, and accurate assessment tool based on clinical evaluation on several domains such as diseases, functioning, and cognition [1]. However, the need of in-person evaluation makes the CFS possibly prone to interrater bias and not always a priority in settings that need to add resources for bedside assessment. Alternatively, automated frailty scores based on readily available electronic health records (EHRs) or administrative claims data are increasingly used as frailty screening tools. Examples include the Hospital Frailty Risk Score (HFRS) calculated based on 109 International Classification of Diseases, Tenth Revision (ICD-10) codes [2], and electronic frailty indices (eFIs) constructed based on the widely validated deficit accumulation model [3, 4]. These scores are generally proven to be valid prognostic tools for predicting mortality, yet they are usually limited to country-specific settings (e.g., the eFI by Clegg et al. is calculated based on the Read codes used in the UK primary care [3], which may not be applicable to other health systems). Testing whether and how automated frailty scores can be applied in other populations and health systems is therefore essential before they can be widely implemented.

**Figure 1:**
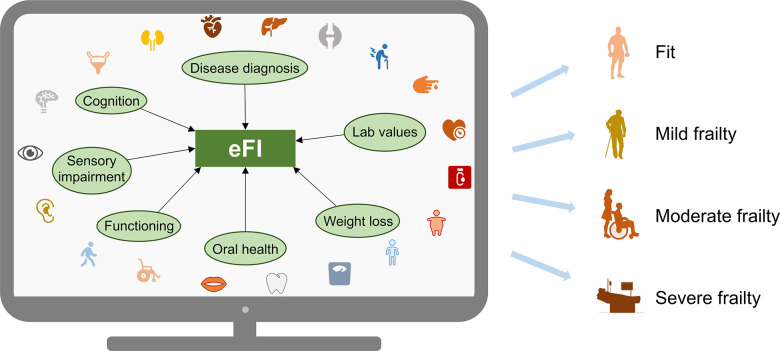
**Schematic diagram illustrating construction of an electronic frailty index (eFI).** Theoretically, any age-related diseases, signs, symptoms, disabilities, and lab values that are available within the electronic health records can be used for constructing the eFI. It is calculated as the sum of deficits divided by the total number of non-missing items (generating a score ranging from 0 to 1) and is usually categorized into three to four groups, where a higher level of frailty is associated with increased risk of mortality.

As another example of the deficit accumulation model, we recently developed an eFI for geriatric patients in Stockholm, Sweden [5], which largely follows the principles of the US eFI model by Pajewski et al. [4]. The Swedish eFI is constructed using 48 items from disease diagnoses (ICD-10 codes), signs and symptoms (e.g., activity limitation, sensory impairment, previous falls, incontinence, oral health), and laboratory and anthropometric measures (e.g., blood pressure, hemoglobin, body mass index) (Figure 1). As expected, the Swedish eFI is strongly associated with mortality outcomes [5] and also has value in risk stratification in COVID-19 patients [6]. For instance, compared to relatively robust patients (eFI ≤0.15), severely frail patients (eFI >0.25) had 32.8 times higher odds of in-hospital mortality (95% confidence interval [CI] 14.7–93.3), 9.8 times higher risk of 30-day mortality (95% CI 7.3–13.2), and 5.8 times higher risk of 6-month mortality (95% CI 5.0–6.8) after adjusting for age and sex [5]. More importantly, the eFI has several advantages over the already available measures (e.g., CFS, HFRS). Firstly, compared to the CFS which has a high proportion of missingness in our data (~60%, mainly due to variations in data collection practice across hospitals), the eFI is based on routinely collected clinical data and has a significantly lower missingness rate (~30%). It can thus potentially reduce physicians’ time and burden of performing a bedside frailty assessment. Secondly, among all the frailty and comorbidity measures that we analyzed (i.e., eFI, CFS, HFRS, and Charlson Comorbidity index), we found that the eFI had the highest discriminative ability for mortality, such that a model including eFI, age, and sex yielded an area under the curve of 0.81 for predicting in-hospital mortality. This finding supports the potential of the eFI as a clinically useful risk stratification tool. Thirdly, the eFI is flexible such that a variety of deficit items available in the EHR can be incorporated in it, as long as the items cover a wide range of physiological systems (i.e., adhering to the multidimensional nature of the deficit accumulation model) (Figure 1). It is also generalizable to several patient groups, including COVID-19 patients [6].

As highlighted in previous studies, it is often impossible to meet all guideline recommendations in clinical practice, mostly because of a lack of time [7]. Therefore, the possibility to get a good frailty scoring based on the already collected data and without the need of extra time for assessment is very promising. Moreover, as frailty is becoming a more important parameter to be considered in clinics outside geriatrics, such as surgery, cardiology, and orthopedics [8], there is also an increasing need of automatic frailty tools in various settings. However, some remaining issues regarding the clinical utility of the eFI are yet to be addressed. To enrich the eFI scores, we need an easy way to get access to all the available data for each person, especially data from primary care. We have already built on a liaison with the EHR system provider, with an aim to incorporate the eFI in the system that is used across primary, secondary, and tertiary care providers in Sweden. Ideally, the eFI can be calculated and updated during each visit, thus capturing both the underlying vulnerability and current illnesses. Another issue to be further studied is whether the eFI has good predictive ability on adverse outcomes other than mortality. For instance, we observed relatively poor discriminative performance of the eFI for 30-day readmission [5]. We are currently examining the association of the eFI with longer-term readmission, healthcare utilization, and other health outcomes in larger datasets, which will hopefully improve our understanding on the wider applications of the eFI. Finally, while the eFI could serve as a frailty screening tool, the effectiveness of frailty screening in the first place, such as whether the eFI could aid in clinical decision-making and improve patient outcomes, is still less clear and warrants further investigation.

In summary, automated frailty scores based on the almost real time-collected patient data from EHRs or similar databases are simple and valuable screening tools that do not require additional work from clinicians. Building on the deficit accumulation model, the eFI specifically captures the multidimensional concept of frailty and is a flexible measure that can potentially be applicable across different settings and patient groups. We encourage more efforts to adapt and test the utility of similar automated frailty scores in other health care settings.
